# UNDERSTANDING THE RELATIONSHIP BETWEEN COPING STYLES AND DEATH ANXIETY IN OLDER ADULTS

**DOI:** 10.1093/geroni/igac059.2524

**Published:** 2022-12-20

**Authors:** Juliamaria Coromac-Medrano, Amber Watts, Christian Sinclair, Ilana Engel

**Affiliations:** University of Kansas, Lawrence, Kansas, United States; University of Kansas, Lawrence, Kansas, United States; University of Kansas Medical Center, Kansas City, Kansas, United States; University of Kansas, Lawrence, Kansas, United States

## Abstract

Research suggests that death anxiety stems from fear of pain, worry about loved ones, and uncertainty about what comes after death. Understanding the relationship between coping styles and attitudes towards death in older adults may help identify individuals who need support with death anxiety. This study explored the relationships between coping styles (active, disengaged, social) and death anxiety (fear, avoidance). We used the Death Attitude Profile Revised and three subscales from the Brief Coping Orientation to Problems Experienced (COPE) Inventory. We conducted linear regressions to determine which coping styles were associated with fear of death and death avoidance. In post-hoc analyses, we investigated the role of spirituality-based coping as a two-item subscale from the active coping scale. All models controlled for age, sex, marital and educational status. The sample included 87 community-dwelling older adults (Mage=72.72 (SD=5.88); 56.32% female; 86.21% White). Higher levels of disengaged coping were significantly associated with greater fear of death and death avoidance (p < .05). Use of social support coping was significantly associated with less fear of death (β = -.10, p < .05). Spirituality-focused coping was associated with lower death avoidance (p < .05). Disengaged coping may indicate higher death anxiety, whereas spirituality and social support coping strategies may indicate lower death anxiety. Our findings have implications for identifying individuals in need of extra support during critical points in the healthcare process. They may also inform design and implementation of psychosocial interventions for communication about healthcare goals in the context of serious or terminal illness.

